# Brain disorder prediction with dynamic multivariate spatio-temporal features: Application to Alzheimer’s disease and autism spectrum disorder

**DOI:** 10.3389/fnagi.2022.912895

**Published:** 2022-08-30

**Authors:** Jianping Qiao, Rong Wang, Hongjia Liu, Guangrun Xu, Zhishun Wang

**Affiliations:** ^1^Shandong Province Key Laboratory of Medical Physics and Image Processing Technology, School of Physics and Electronics, Shandong Normal University, Jinan, China; ^2^Department of Neurology, Qilu Hospital of Shandong University, Jinan, China; ^3^Department of Psychiatry, Columbia University, New York, NY, United States

**Keywords:** deep learning, dynamic functional connectivity, fMRI, multivariate, spatio-temporal features

## Abstract

The dynamic functional connectivity (dFC) in functional magnetic resonance imaging (fMRI) is beneficial for the analysis and diagnosis of neurological brain diseases. The dFCs between regions of interest (ROIs) are generally delineated by a specific template and clustered into multiple different states. However, these models inevitably fell into the model-driven self-contained system which ignored the diversity at spatial level and the dynamics at time level of the data. In this study, we proposed a spatial and time domain feature extraction approach for Alzheimer’s disease (AD) and autism spectrum disorder (ASD)-assisted diagnosis which exploited the dynamic connectivity among independent functional sub networks in brain. Briefly, independent sub networks were obtained by applying spatial independent component analysis (SICA) to the preprocessed fMRI data. Then, a sliding window approach was used to segment the time series of the spatial components. After that, the functional connections within the window were obtained sequentially. Finally, a temporal signal-sensitive long short-term memory (LSTM) network was used for classification. The experimental results on Alzheimer’s Disease Neuroimaging Initiative (ADNI) and Autism Brain Imaging Data Exchange (ABIDE) datasets showed that the proposed method effectively predicted the disease at the early stage and outperformed the existing algorithms. The dFCs between the different components of the brain could be used as biomarkers for the diagnosis of diseases such as AD and ASD, providing a reliable basis for the study of brain connectomics.

## Introduction

Hemodynamics is inextricably linked to neuronal activation. Previous studies have demonstrated that the interactivity among brain regions is a powerful tool to study the working mechanisms of the brain (Heuvel and Pol, 2010). Resting-state functional magnetic resonance imaging (rs-fMRI) characterizes the activity state of the brain in the absence of task or stimulation. The hemodynamic changes are measured by analyzing magnetic resonance imaging (Biswal et al., 1995). The rs-fMRI accurately captures the functional impairment that occurs in AD (Collaborators, 2019; Cummings et al., 2019) and autism spectrum disorder (ASD) (Heinsfeld et al., 2018; Bajestani et al., 2019), which are important changes in functional connectivity (FC) in the brain. AD is a neurodegenerative disease with the main onset period being old age and pre-geriatric age. The neuropathological changes of AD are reflected in neurofibrillary tangles and amyloid deposits. The main manifestations are cognitive decompensation and non-cognitive neuropsychiatric symptoms (Association, 2013, 2015). Numerous studies have shown that patients with AD have developed severe neurodegeneration in the brain and the degeneration is irreversible (Li et al., 2012; Zhang et al., 2012; Wee et al., 2013). Patients may experience significant atrophy in multiple brain regions that affect all functions. Imaging studies suggest that AD is a “disconnectedness syndrome” that manifests as abnormalities in resting-state functional connectivity of brain networks. There are early stages of mild cognitive impairment (MCI) and late stages of MCI before the development of AD. The early diagnosis of AD is of great significance. On the contrary, ASD is a serious developmental disorder mostly presented in childhood and adolescence (Goldani et al., 2014), which is mainly reflected in deficits in communication and language skills. Previous brain imaging studies have found that the connectivity of different regions of the brain and neural networks is altered in infants and children with autism (Linke et al., 2020). The causes of ASD are complex which may be related to the interaction of various genetic and environmental factors. There is no consistent research result so far. ASD is a serious threat to the physical and mental health of children which poses a serious burden to families and society. Connections between resting-state networks (RSNs) reflect functional interactions between different brain networks. The abnormal connections in AD and ASD could serve as features for early diagnosis of these diseases. Therefore, computer-assisted diagnosis is helpful for the discovery of valid biomarkers and better understanding of the disease mechanisms.

The traditional methods for fMRI brain networks consist of two categories: model-driven and data-driven based methods. Specifically, the distribution and integration of functions are obtained by correlating the time series between different regions of the brain in the model-driven approaches such as seed-based analysis (SBA) (Jiang et al., 2004; Buckner et al., 2009). However, this method requires the delineation of the fixed area which makes it impossible to fully represent the information contained in the data. Independent component analysis (ICA) (Ibrahim et al., 2020) is a powerful data-driven method (Beckmann et al., 2005; Smith et al., 2009) that aims to decompose a data matrix into products of spatial patterns and corresponding time series without *a priori* knowledge or preexisting assumptions. The spatial independent brain networks acquired by ICA could capture the complex nature of the fMRI time course (Turner and Twieg, 2005; Calhoun and Adali, 2006). The ICA-based model identifies potential sources in whole-brain voxels with the same time course and groups them into the same component in the spatial domain compared with the regions of interest (ROI)-based approach. Therefore, each component can be considered as a measure of the degree of correlation between voxels and components.

ICA is gaining popularity as a powerful tool for biomedical data analysis, especially in biomedical imaging such as fMRI. ICA reduced the number of functional connectivity correlations and increased the strength of these correlations in an fMRI study of epilepsy patients (Parsons et al., 2020). Another study proposed a group ICA (GICA) model to describe group-level inferences of fMRI data (Calhoun et al., 2001). Extended ICA was used to extend fMRI data from a single subject to a group of subjects (Svensen et al., 2002). The results showed that GICA can extract relevant components without any *a priori* information about fMRI and identify common components of the group in the analysis of group data. Moreover, ICA is used for signal decomposition and feature extraction as an integral part of machine learning (ML) disease diagnosis. Study has shown that the selection of the subset ICA features can significantly improve the performance of the classifier compared to ML alone (Aziz et al., 2016). Researchers combined ICA with random forest (RF) algorithm for predicting whether a subject was viewing a video, listening to an audio, or at rest (Xie et al., 2017). ICA was applied to multisite fMRI data, and six RSNs were seen as features to identify ASD patients (Sun et al., 2021). A hierarchical partner matching ICA method was proposed in our previous work in which the directed acyclic graph neural network was applied for AD classification (Qiao et al., 2018). Although ICA has been used to the analysis and classification of fMRI data, the existing methods assumed that brain activity was quiescent over a short period of time which ignored the underlying temporal signal characteristics (Hutchison et al., 2013).

Temporal dynamics of the fMRI data refers that the functional connectivity varies over time in brain activity (Miller et al., 2016; Fu et al., 2019). The most popular approach for dynamic FC is that different regions are partitioned based on a spatial scale, and the correlation between two regions is computed within the sliding time window (Preti et al., 2017). The whole-brain dFCs were calculated by using a sliding window technique, and SVM was used to classify normal individuals, schizophrenia (SZ), schizoaffective disorder (SAD), and bipolar disorder with psychosis (BPP) (Du et al., 2020). A graph convolution-based LSTM (GC-LSTM) network was proposed to extract valid disease-related features from the dFC for AD classification (Xing et al., 2021). Another study calculated the whole-brain time-varying connectivity matrix based on 116 ROIs in the automatic anatomical labeling (AAL) template, and a bidirectional LSTM network (Full-BiLSTM) was used to classify the output of NC and MCI (Yan et al., 2018). A convolutional neural network (wck-CNN) was designed containing a weighted correlation sum to sequentially extract local, global, and temporal features from the constructed dFC for AD, NC, early MCI (EMCI), and late MCI (LMCI) classification (Jie et al., 2020). However, the construction of dynamic connectivity matrix relies on the delineated ROIs of the specific templates. The different templates may lead to uncertain classification results and increase the variability of the pipeline. Moreover, certain brain voxels may not be associated with disease, and the use of whole-brain ROIs for classification runs the risk of increasing redundant features.

Recently, researchers have applied ICA in combination with dFC in the analysis of fMRI data. For example, ICA and dFC were used to explore brain remodeling, which indicated significant differences in functional brain remodeling between two groups, with higher functional brain remodeling in patients with ASD and SZ (Fu et al., 2021). Moreover, grouping dynamic connections between ICs and clustering them into different states have been proposed to reveal the flexibility of functional coordination between different nervous systems (Allen et al., 2014; Calhoun et al., 2014). The dynamic brain networks among ICs were clustered into multiple states for the classification of SZ, ASD, and NC by using the linear discriminant analysis (Rabany et al., 2019). However, the before-and-after time correlation of dynamic processes was not considered in these methods. The missing dynamical characteristics of the time dimension of the fMRI data may made low classification performance.

In this paper, we proposed a dynamic ICA combined with LSTM network (dICA-LSTM) approach for early diagnosis of the AD and ASD which investigated the feasibility of dynamic temporal properties of spatially independent brain function sub networks as potential disease biomarkers. Specifically, the spatial independent component analysis (SICA) method was applied to the rs-fMRI data to obtain several components that were spatially maximally independent of each other. The sliding window method was used to obtain the dynamic functional connectivity (dFC) status between different ICs. Then, the dynamic characteristics of the functional connectivity values between each pair of ICs were obtained as features. Finally, the features with temporal characteristics were used as the input to the LSTM network for disease prediction. The highlights of the method include the following: (i) The ICA method is used to partition the spatial domain of the brain map automatically and capture the multivariate features which are more targeted than that from the traditional ROI template. The data-driven approach overcomes the dependence on established templates while avoiding the self-contained nature of model-driven approaches. (ii) The dynamic fMRI specifically depicts the process of functional connectivity between spatial patterns in the brain over time, allowing even small changes that occur within a short period of time to be identified. The dynamic properties in this method solve the problem of the potential kinetic properties ignorance in the traditional ICA methods and static FC studies, providing methodological support for parsing the higher cognitive functions of the human brain and mapping the connectivity of the human brain. (iii) The LSTM network is specific to temporal signals in the field of deep learning. We applied the LSTM to fit with the extracted dynamic temporal features which solve the problem that traditional classifiers are insensitive to temporal signals. We applied the method to two separate databases, Alzheimer’s Disease Neuroimaging Initiative (ADNI) and Autism Brain Imaging Data Exchange (ABIDE).^1^ The ICA-based LSTM network illustrates the existence of temporal dynamic interpretability of functional connectivity among human brain networks and that temporal signals could effectively characterize individual disease states.

## Materials and methods

**FIGURE 1:**
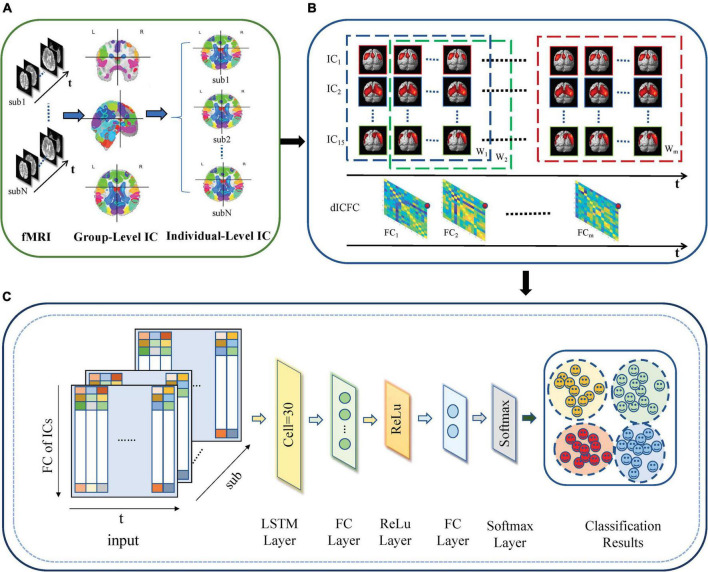
dICA-LSTM pipeline diagram. **(A)** Group ICA decomposes resting-state data into 20 components at the group level. The individual-level independent components are estimated by the back reconstruction. **(B)** Extracting time series for 15 spatially independent components after removing noise components. A sliding window with a window width of 50TR and a step size of 1TR splits the time series into *m* windows (W_1_–W_*m*_). The correlation of the time series within each window (FC_1_–FC_*m*_) is calculated. **(C)** The dynamic functional connections between independent components are used as the input of the deep learning network. The network includes an LSTM layer, two fully connected layers, a ReLu layer, a softmax layer, and a classification layer.

The proposed dICA-LSTM disease classification framework based on rs-fMRI is composed of three modules (shown in Figure 1): individual-level space components, construction of dynamic functional connections between independent components, and LSTM network models.

### Preprocessing

The standard preprocessing and feature matrix construction methods were used for all rs-fMRI data in ADNI and ABIDE datasets to achieve reliable and reproducible solutions. Data preprocessing procedure was carried out by using the Data Processing Assistant for Resting-State fMRI (DPARSF) (Chao-Gan and Yu-Feng, 2010) and Statistical Parametric Mapping (SPM12) toolboxes. First, the first 10 volumes of resting-state fMRI data were removed to prevent the occurrence such as signal instability at the beginning of the scan and the maladjustment to noise. Second, the slices of the fMRI data were acquired at different times within the repetition time (TR). Therefore, temporal layer corrections were taken for all time points. Third, the motion correction was performed to ensure the brain image move to the same position. The middle slice was used as the reference slice for slice timing correction. The subjects with head movements larger than 2 mm and rotation angles larger than 2° were excluded to ensure the reliability of the data. Forth, the images were normalized into standard Montreal Neurological Institute (MNI) EPI space and resliced to 3 mm × 3 mm × 3 mm voxels. Finally, the images were smoothed with a 4-mm full-width at half-maximum (FWHM) Gaussian kernel. A low-frequency filter of 0.01–0.08 Hz was used to reduce the influence of extremely low-frequency and high-frequency physiological noise.

### Extraction of spatial components

The SICA can identify event-related and spatially independent sub networks of brain functions in multivariate data without restricting the time domain. In this study, the 10-fold cross-validation was performed in the ICA process. Specifically, all subjects were divided into 10-fold. Onefold was used as the testing set, and the remaining ninefold was used as the training set. The process was repeated 10 times until each fold was used as the testing set. At the training stage, the SICA in Group ICA Of fMRI Toolbox (GIFT) (Allen et al., 2012) was performed on the training subjects. The training data were subjected to principal component analysis (PCA) dimension reduction followed by ICA with the Infomax algorithm. Finally, the RSNs with physiological significance were obtained after removing the noise components. At the testing stage, the PCA downscaling and Infomax algorithms were applied to the testing fMRI data. Then, the same ICA parameters as the training set were applied to obtain the RSNs of the test subjects. Finally, the average of the ten testing loops was taken as the final result.

**FIGURE 2:**
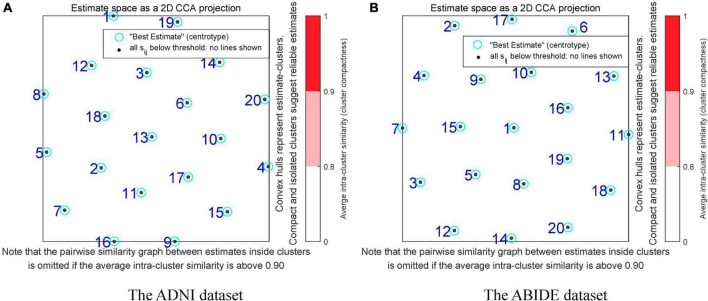
Reliability assessment in the independent component decomposition process of two datasets. **(A)** The reliability assessment of the ADNI dataset. **(B)** The reliability assessment of the ABIDE dataset. The black dots represent the estimated ICs in single run. The numbers of the clusters are labeled by the stability index, and minimum and maximum cluster size. The compact and isolated clusters indicate the reliable estimation. The different colors in the right bar indicate the similarity between different clusters. The darker red color (0.9–1) indicates the higher similarity.

The GICA methods consisted of three parts: data reduction, independent component computation, and reverse reconstruction. First, the PCA was used to reduce the data for the dimension reduction. Here, the number of principal components was reduced to 20 by using two times of PCA based on 95% variance of the original data. Next, ICA with the Infomax algorithm was performed to decompose the data into 20 independent components and obtain group-level independent components (GLIC). Specifically, the ICASSO algorithm was used to run the ICA decomposition 10 times to ensure the reliability and stability of the evaluated components (Himberg et al., 2004). The reliability evaluation results of the independent decomposition method are shown in Figure 2. The black dot represented the estimated ICs in single run. The label number of the cluster was classified by the stability index, and minimum and maximum cluster size. The compact and isolated clusters suggested the reliable estimation. The different colors in the right bar indicated the similarity between different clusters. The darker red color (0.9–1) indicated the higher similarity. The results that there were no red areas in Figure 2 indicated the high independence of the divided components. Finally, we performed group information-guided ICA (GIG-ICA) (Salman et al., 2018) for the reverse reconstruction to obtain spatial maps and time courses for individual-level independent components (ILIC).

### Construction of dynamic functional connectivity matrix at the independent component analysis level

**FIGURE 3:**
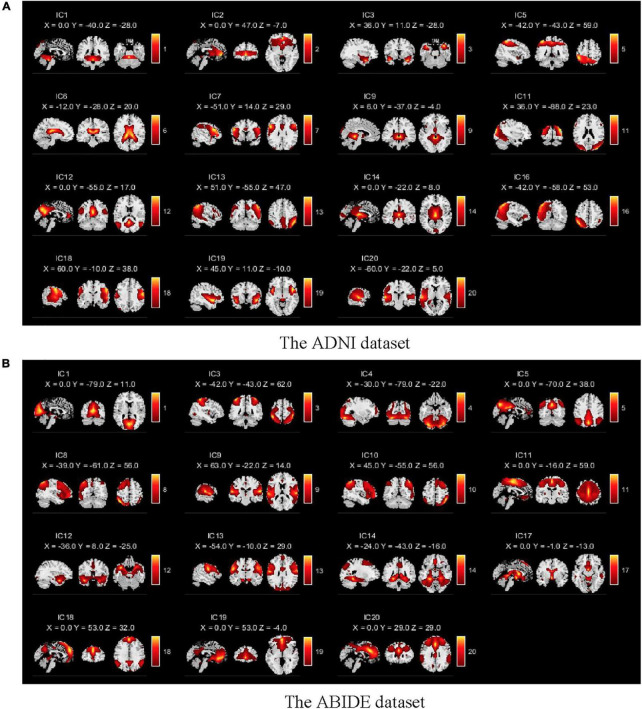
Spatial components obtained by the GICA for two databases. **(A)** The spatial components of the ADNI dataset. **(B)** The spatial components of the ABIDE dataset. There are 15 meaningful independent components in the GICA process after eliminating obvious noise components.


We selected 15 meaningful RSNs after removing the noise components from the estimated 20 stable ICs (shown in Figure 3). The time series of the RSNs were post-processed including detrending, regression, and filtering. Then, the dFC matrix was constructed by a sliding window with a window length of 50TR and a step size of 1TR. Specifically, let $T_{m}=\left[T_{m}^{1},T_{m}^{2},\ldots \,\ldots ,T_{m}^{w}\right]$ be the sub-time series of the *m*th subject, where *w* was the number of windows, $T_{m}^{w}$ represented the *w*th sub-time series of the *m*th subject which was a two-dimensional matrix of *50 (Width of the window)*×*15 (Number of the RSN)*. Then, let $R_{m}=\left[R_{m}^{1},R_{m}^{2},\ldots \,\ldots ,R_{m}^{w}\right]$ denotes the sub-connection matrix obtained by applying Pearson’s correlation to *T*_*m*_. The $R_{m}^{w}$ was the *w*th sub-functional connectivity matrix of the *m*th subject, where the size of $R_{m}^{w}$ was *15*×*15*. Here,

(1) $$R_{m}^{w}=\{\begin{array}{c} corr\left[T_{m}^{w}\left(:,p\right),T_{m}^{w}\left(:,q\right)\right],if\left(p\neq q\right);\\ 1,if\left(p=q\right). \end{array}$$

where *corr* was the Pearson correlation coefficient. If $T_{m}^{w}\,\left(:,i\right)$ and $T_{m}^{w}\,\left(:,j\right)$ were denoted as variables *A* and *B*, respectively. We kept the sign of the Pearson correlation coefficient, then


(2) $$\mathrm{corr}\,\left[A,B\right]=\frac{\sum_{k=1}^{n}\frac{\left(A_{k}-E\,\left(A\right)\right)}{\sigma_{A}}\,\frac{\left(B_{k}-E\,\left(B\right)\right)}{\sigma_{B}}}{n}$$

where *E*(*A*)*andE*(*B*) were the means of the variables *A* and *B*. The σ_*A*_*and*σ_*B*_ were the standard deviations of *A* and *B*. Since $R_{m}^{w}$ was the real symmetric array, the upper triangular elements were selected to avoid feature redundancy which was spread as the one-dimensional vector, denoted by $X_{m}^{w}=t\,r\,i\,u\,\left(R_{m}^{w}\right)$. Then, the dynamic features of each subject at the ICA level were represented by the *105* elements in $X_{m}^{w}$. For instance, the dimension of the feature was 105 and the dynamic feature matrix of the *m*th subject could be represented as $X_{m}=\left[X_{m}^{1},X_{m}^{2},\ldots \,\ldots ,X_{m}^{w}\right]$ with size of 105×*w*, where w referred to the number of windows in the sliding process, that was, the change of the feature at w time points. In this way, the time-varying multidimensional features of each subject were obtained. Let the set of subjects was denoted as *S* = {*S*_1_;*S*_2_;……;*S*_*N*_}, where *N* represented the number of subjects. Then, the feature characteristics of all subjects were combined as *X* = [*X*_1_;*X*_2_;……;*X*_*N*_] which was used as the input of the subsequent LSTM network.

### Construction of dynamic functional connectivity matrix at the regions of interest level

The dFC matrix at the ROI level was also computed to cross-verify the effectiveness of temporal properties of fMRI and the validity of ICA. Specifically, the AAL template (Tzourio-Mazoyer et al., 2002) was used to divide the preprocessed images into 116 brain regions. The time series of all voxels within each brain region were averaged. Then, the time series of each region was segmented using the same window width and step size as that used at the ICA level. Subsequently, we computed the functional connectivity matrix between different brain regions within each window and its upper triangular elements. Since the number of brain regions was different from the number of RSNs obtained by ICA, the dimension of the feature vector for each subject at the ROI level was $Y_{m}^{w}=6670$. Then, the dynamic feature matrix of the *m*th subject was denoted as $Y_{m}=\left[Y_{m}^{1},Y_{m}^{2},\ldots \,\ldots ,Y_{m}^{w}\right]$ with the size of 6670×*w*, where w referred to the number of windows in the sliding process and the dimension of the feature was *6670*. Finally, the features of all subjects were combined as *Y* = [*Y*_1_;*Y*_2_;……;*Y*_*N*_].

### Extraction of the static features

The static functional connectivity matrices at the ICA and ROI level were also conducted to illustrate the reliability of dynamic features as potential biomarkers. The specific process was expressed as a direct correlation analysis of the obtained time series at the ICA and ROI levels, thus obtaining the functional connectivity matrix of the entire time course as features without taking a sliding window. Then, the upper triangular elements of the functional connectivity matrix were tiled into a one-dimensional vector as the feature vector. Finally, two representative classifiers, SVM and RF, were applied for classification. Compared with the dynamic features based on sliding windows, the features lacked information about the time dimension which was therefore called static features.

### Classification with the long short-term memory network

The LSTM network has mostly been applied to the prediction of signals with strong temporal characteristics such as speech and text but is little used in neuroimaging (Atila and Sengur, 2021; Peng et al., 2021). The aim of this study was to investigate the potential power of the temporal hierarchy for the diagnosis of AD and ASD. Therefore, the LSTM network was utilized to capture temporal information for the extraction of the dynamic features.

The recurrent neural network (RNN) is prone to stop learning when the gradient disappears during backpropagation and thus has short-term memory. As the variant of RNN, the LSTM network overcame this problem. The LSTM is composed of several identical components, called hidden cells, where multiple hidden cells are connected first and last. It can pass and forget information through gate structures and cell states. The information in all cells is sent to the fully connected layer to obtain the final diagnostic results. In this study, the structure of the proposed network includes the input layer, LSTM layer, fully connected layer, activation function layer, softmax layer, and classification output layer. The LSTM network was a multiple-input single-output model whose input was the product of the feature count time-step matrices and the output was a categorical type categorical column vector. The architecture of the LSTM network in the study consisted of 30 hidden cells, and each cell is made up of three controlled structures: input gate, forgetting gate, and output gate. The LSTM layer maintained the values that were determined in the previous period by these gates which controlled the data transmission in the cell (Hochreiter and Schmidhuber, 1997). The mechanism of the operation in each cell was written as follows:


(3) $$h\left(t\right)=\sigma \left\{\omega_{o}\left[h\left(t-1\right),X\left(t\right)\right]+b_{o}\right\}\cdot \tanh[C\left(t-1\right)\cdot f\left(t\right)$$

$$+i\left(t\right)\cdot \tilde{C}\left(t\right)]$$

The forgetting gate was structured as the single-layer neural network as shown in equation (4). If the output was one, the forgetting gate was activated.


(4) $$f\,\left(t\right)=\sigma \,\left(\omega_{f}\cdot \left[h\,\left(t-1\right),X\,\left(t\right)\right]+b_{f}\right)$$

The input gate consisted of two parts including the current sequence and the memory sequence which were represented by the following equations:


(5) $$i\,\left(t\right)=\sigma \,\left(\omega_{i}\cdot \left[h\,\left(t-1\right),X\,\left(t\right)\right]+b_{i}\right)$$

(6) $$\tilde{C}\,\left(t\right)=\tanh\,\left(\omega_{c}\cdot \left[h\,\left(t-1\right),X\,\left(t\right)\right]+b_{c}\right)$$

where *X*(*t*)*andh*(*t*) were the input and output of the current cell. The *C*(*t*−1) and *h*(*t*−1) were the state and output of the previous cell. The σ denoted the logical sigmoid function. The ω_*o*_,ω_*f*_,ω_*i*_,*and*ω_*c*_were different weighting factors. The *b*_*o*_,*b*_*f*_,*b*_*i*_,*andb*_*c*_ were corresponding to different biases.

## Experimental datasets and evaluation criteria

### Datasets

The rs-fMRI data from two independent databases ADNI and ABIDE (Di Martino et al., 2014) were applied to evaluate the effectiveness of the proposed method in this study. The data in the ADNI database came from two scanners including Philips Medical Systems and Siemens. The scanning parameters of Philips were as follows: field strength = 3T, repetition time = 3,000 ms, echo time = 30 ms, flip angle = 80°, matrix = 64 × 64, slice thickness = 3.3 mm. The scanning parameters of Siemens were as follows: field strength = 3T, repetition time = 3400 ms, echo time = 12 ms, flip angle = 90°, matrix = 64 × 64, slice thickness = 4 mm. The data with excessive head movement were excluded, and we applied 121 patients with AD, 161 healthy controls, 61 patients with EMCI, and 49 patients with LMCI in this study. The population consisted of middle-aged and older adults aged 55–90 years. On the one hand, we performed the two-by-two classification task for four populations. Therefore, the subjects were divided into six cases including AD vs. NC, AD vs. EMCI, AD vs. LMCI, NC vs. EMCI, NC vs. LMCI, and EMCI vs. LMCI. On the other hand, multi-class classification was performed for the four stages of the AD disease. The ABIDE dataset is a large-scale assessment dataset of the intrinsic brain structure of autism. In this study, the data were scanned by Siemens. The scanning parameters were as follows: field strength = 3T, repetition time = 2,500 ms, echo time = 30 ms, flip angle = 90°, matrix = 64 × 64, slices = 36. The subjects from the ABIDE database including 82 patients with ASD and 75 healthy controls were dichotomized. The specific demographic information could be found in Table 1.

**TABLE 1:** Demographic information of subjects in the ADNI and ABIDE databases.

Dataset	ADNI				ABIDE	
Group	AD	NC	EMCI	LMCI	ASD	NC
Number	121	161	61	49	82	75
Age	74.6 ± 7.76	74.81 ± 7.65	72.12 ± 7.22	71.11 ± 7.7	19.53 ± 7.08	16.12 ± 5.71
M/F	64/57	70/91	25/36	26/23	74/8	60/15

### Evaluation criteria

The 10-fold cross-validation was utilized to evaluate the performance of the model. The data were randomly divided into 10-fold. In each loop, the 9-fold was applied for training, while the remaining 1-fold was used for testing. The average of the ten loops was used as the final classification result. Four evaluation metrics were used in the experiments including accuracy (ACC), sensitivity (SEN), specificity (SPE), and F1 score (F1) (Barandela et al., 2003).


(7) $$\mathrm{ACC}=\frac{\mathrm{TP}+\mathrm{TN}}{\mathrm{TP}+\mathrm{FP}+\mathrm{TN}+\mathrm{FN}}$$

(8) $$\mathrm{SEN}=\frac{\mathrm{TP}}{\mathrm{TP}+\mathrm{FN}}$$

(9) $$\mathrm{SPE}=\frac{\mathrm{TN}}{\mathrm{TN}+\mathrm{FP}}$$

(10) F-1⁢score=2⁢precision*⁢recallprecision+recall*

where the true positive (TP) was the number of correctly predicted patients in the patient sample. The true negative (TN) was the number of correctly predicted in a normal control. The false positive (FP) was the number of normal controls predicted to be patients. The false negative (FN) was the number of patients predicted to be normal.

We also performed multi-classification to predict the AD, LMCI, EMCI, and NC on the ADNI database. The Kappa coefficient and Jaccard similarity score were introduced as evaluation metrics in the quadratic classification.


(11) $$\mathrm{Kappa}=\frac{p_{o}-p_{e}}{1-p_{e}}$$

(12) $$\mathrm{Jaccard}\,{\text{y}}_{\text{test}}\,{\text{y}}_{\text{pred}}=\frac{\left|{\text{y}}_{\text{test}}\,\cap {\text{y}}_{\text{pred}}\right|}{\left|{\text{y}}_{\text{test}}\,\cup {\text{y}}_{\text{pred}}\right|}$$

(13) $$p_{e}=\frac{\sum s^{*}\,c}{{\text{T}}^{2}}$$

where *p*_*o*_ was the overall classification accuracy, *y*_*test*_ was the set of sample labels, *y*_*pred*_ was the set of prediction results, *s* was the sample size of category, *c* was the number of samples correctly classified, and *T* was the total number of samples.

## Results

### Classification results of dynamic features

**FIGURE 4:**
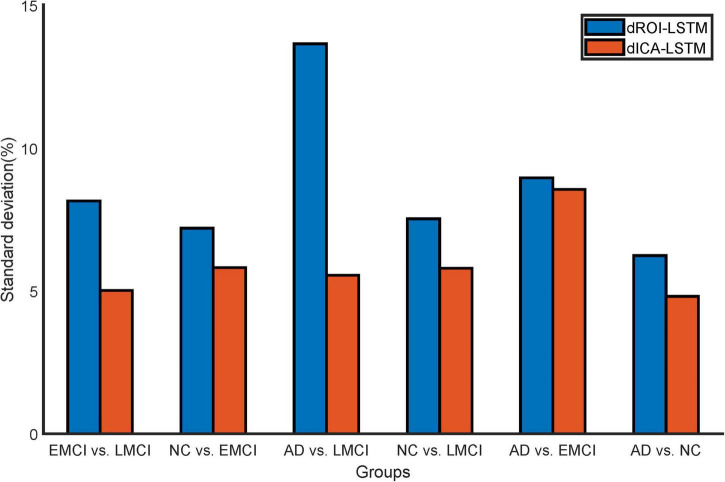
Standard deviation comparison of classification accuracy in 10-fold cross-validation for the dynamic ROI and dynamic ICA methods. The standard deviations of the accuracy with dynamic ICA methods are smaller than that of the dynamic ROI methods during cross-validation.

**FIGURE 5:**
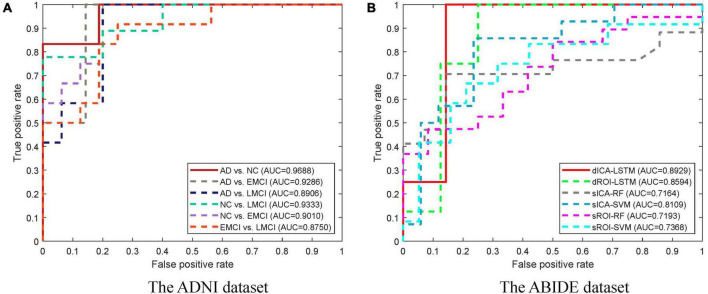
ROC curves for different groups and different methods. **(A)** The ROC curves for different groups of the ADNI dataset. The highest area under the curve (AUC) is obtained in the AD vs. NC task. **(B)** The ROC curves for different methods of the ABIDE dataset. The dICA-LSTM method obtained the best AUC in the classification task.

The classification results based on dynamic features at the ROI level and the ICA level are shown in Table 2. For the ADNI dataset, we performed two-by-two classification of four types of subjects and obtained six sets of classification tasks which were AD vs. NC, AD vs. EMCI, AD vs. LMCI, NC vs. EMCI, NC vs. LMCI, and EMCI vs. LMCI. For the ABIDE dataset, we distinguished ASD patients from healthy subjects. It could be clearly seen that the classification performance of the proposed dynamic ICA features method was significantly improved compared to that of the dynamic ROI features method on both ADNI and ABIDE datasets. Specifically, the accuracy of 96.14% was obtained in AD vs. NC with sensitivity of 98.13%, specificity of 93.53%, and F1 score of 96.73%. The accuracy in ASD vs. NC achieved 84.69%. Furthermore, both the features at the ICA and ROI levels exhibited the best result in AD vs. NC and the relatively poor results in the EMCI vs. LMCI which was consistent with the previous studies (Wee et al., 2019). We also found that the results of the ROI dynamic method had more fluctuation during the cross-validation process, while the results of ICA dynamic method had smaller standard deviation (shown in Figure 4). This might be due to the fact that the brain sub networks obtained by ICA maximized the functional homogeneity within the sub network while the heterogeneity between sub networks makes the time course more reliable. Furthermore, the proposed dynamic ICA-LSTM method achieved 81.9% Kappa coefficient, 87.35% Jaccard coefficient, and 87.5% accuracy as shown in Table 3, indicating that the proposed method had good performance for the multi-classification of AD. The receiver operating characteristic (ROC) curves in Figure 5A depicted the classification performance of the six cases on the ADNI dataset, while Figure 5B compared the classification performance of our proposed method with other methods on the ABIDE dataset.

**TABLE 2:** Comparison of the dynamic ROI-level and ICA-level classification performance.

Datasets	Groups	Number	dROI-LSTM				dICA-LSTM			
			ACC (%)	SEN (%)	SPE (%)	F1 (%)	ACC (%)	SEN (%)	SPE (%)	F1 (%)
ADNI	EMCI vs. LMCI	61 vs. 49	89.42	88.31	87.50	88.59	89.97	88.00	91.90	88.35
	NC vs. EMCI	99 vs. 61	90.25	90.25	86.67	93.13	91.32	91.67	91.00	89.76
	AD vs. LMCI	121 vs. 49	88.26	93.10	88.59	80.63	93.53	72.14	99.17	86.27
	NC vs. LMCI	99 vs. 49	89.55	86.00	83.50	94.08	91.95	82.00	97.00	86.19
	AD vs. EMCI	121 vs. 61	87.01	82.36	84.42	86.60	89.56	72.14	98.33	83.33
	AD vs. NC	121 vs. 161	93.82	86.00	89.17	95.96	96.14	98.13	93.53	96.73
ABIDE	ASD vs. NC	85 vs. 72	83.25	96.00	76.87	82.86	84.69	98.13	87.50	86.05

dROI-LSTM, dynamic ROI combined with LSTM network; dICA-LSTM, dynamic ICA combined with LSTM network.

**TABLE 3:** Results of four classification tasks.

Class	Precision	Sensitive	F1	ACC	Kappa	Jaccard
AD	91.67%	100.0%	95.65%	–	–	–
NC	94.12%	94.12%	94.12%	–	–	–
EMCI	71.43%	83.33%	76.92%	–	–	–
LMCI	75.00%	60.00%	66.67%	–	–	–
Avg	83.06%	84.36%	83.34%	87.50%	81.90%	87.35%

### Classification results of static features

**FIGURE 6:**
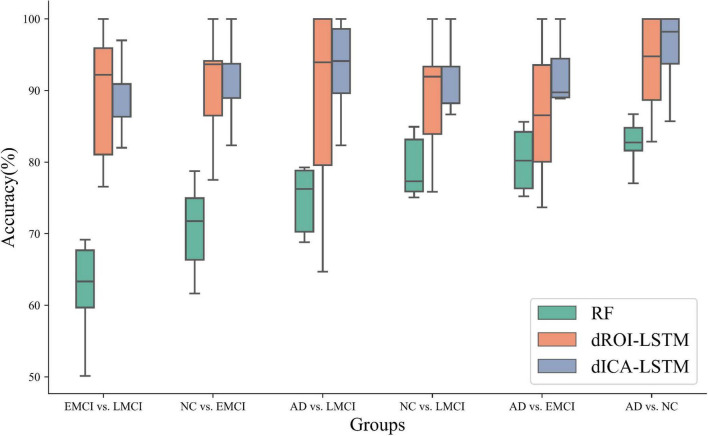
Classification performance comparison of three methods including random forest, dynamic ROI levels, and dynamic ICA levels. The performance of the dynamic features outperforms the static features. Moreover, the performance of the dynamic features at the ICA level outperforms the dynamic features at the ROI level. (RF: random forest, dROI-LSTM: dynamic features at the ROI level combined with LSTM, dICA-LSTM: dynamic features at the ICA level combined with LSTM).

The experimental results of the static features methods are presented in Table 4. The classification performance of static features was lower than that of dynamic features. For static ROI, the classification task for AD and NC performed best in ADNI dataset with an accuracy of 78.33%. Nevertheless, there was still a 15.49% gap relative to the dynamic ROI. For static ICA, the performance was slightly better than that of the static ROI, with the highest accuracy of 82.31%, but there was still a 13.83% gap compared to the ICA dynamic method, as shown in Figure 6 which illustrated the comparison results of dynamic and static cases.

**TABLE 4:** Comparison of the static ROI-level and ICA-level classification performance.

Datasets	Groups	Method	sROI				sICA			
			ACC (%)	SEN (%)	SPE (%)	F1 (%)	ACC (%)	SEN (%)	SPE (%)	F1 (%)
ADNI	EMCI vs. LMCI	SVM	55.63	62.10	65.33	63.58	61.43	64.25	88.76	74.16
		RF	59.05	61.54	55.56	64.00	62.38	79.64	65.83	73.92
	NC vs. EMCI	SVM	65.73	67.98	79.35	63.18	68.91	73.33	78.68	75.97
		RF	69.51	72.85	64.51	62.49	70.84	68.88	82.36	83.14
	AD vs. LMCI	SVM	73.68	76.39	92.11	77.64	74.49	79.38	88.64	83.91
		RF	74.45	75.60	90.23	76.79	74.67	78.40	83.46	85.95
	NC vs. LMCI	SVM	76.31	66.38	77.60	70.24	80.52	79.63	83.20	85.64
		RF	76.80	74.14	85.10	69.86	79.12	78.55	79.68	73.58
	AD vs. EMCI	SVM	75.89	80.60	87.74	87.74	78.30	80.66	91.48	83.50
		RF	75.67	81.98	82.40	81.95	80.22	77.30	96.44	87.53
	AD vs. NC	SVM	76.69	82.50	82.50	83.56	81.01	83.46	95.10	88.42
		RF	78.33	78.33	84.19	83.74	82.31	85.76	85.59	76.84
ABIDE	ASD vs. NC	SVM	72.13	75.62	81.22	78.90	76.98	74.82	84.39	85.61
		RF	63.48	61.60	76.63	63.68	65.77	74.50	65.70	69.41

sROI, static features at the ROI level; sICA, static features at the ICA level.

### Comparison with the existing methods

We compared our method with the previous research studies, and the results are indicated in Table 5. The classification performance of our method exhibited significant improvement over the existing methods on the ADNI dataset. In particular, the ACC built up about 3.39% in EMCI vs. LMCI relative to other methods and 3.24% in the classification tasks of AD and NC. Table 6 has shown that our method had an accuracy rate of 84.69% on the ABIDE database which was advantageous. The results verified that the proposed dynamic ICA functional brain network connections combined with the time-sensitive LSTM network exploited the fMRI spatial-temporal properties, enabling outstanding performance in the AD and ASD prediction.

**TABLE 5:** Comparison with other methods on ADNI database.

References	Number	Methods	Groups	ACC (%)	SEN (%)	SPE (%)	AUC (%)	F1 (%)
Wee et al. (2016)	EMCI29, NC30	Sparse network	NC vs. EMCI	79.66	75.86	83.33	79.20	78.57
Guo et al. (2017)	EMCI33, LMCI32, AD29, NC28	Hyper-network	NC vs. EMCI	72.80	78.25	67.13	–	–
			NC vs. LMCI	78.63	82.54	72.18	–	–
			AD vs. NC	91.60	93.50	90.50	–	–
Shi et al. (2020)	AD67, NC76	FC + LSVM	AD vs. NC	92.9	100	86.67	–	–
Xing et al. (2021)	AD115, NC177	GC-LSTM	AD vs. NC	90.0	91.7	88.6	–	–
Liu et al. (2020)	EMCI29, LMCI18, NC29–	ASBiLSTM	NC vs. EMCI	87.93	96.55	79.31	95.96	–
			NC vs. LMCI	91.49	96.55	83.33	97.24	–
			EMCI vs. LMCI	89.36	93.10	88.89	94.16	–
Yang et al. (2021)	EMCI29, LMCI18, NC29	Fused sparse network	EMCI vs. LMCI	80.85	71.06	–	84.87	–
			NC vs. EMCI	82.76	68.79	–	88.23	–
			NC vs. LMCI	87.23	75.58	–	92.34	–
Song et al. (2021)	EMCI44, LMCI38, NC44	SAC-GCN	EMCI vs. LMCI	86.58	92.10	81.81	94.26	–
			NC vs. EMCI	85.22	90.90	79.54	89.92	–
			NC vs. LMCI	89.02	89.47	88.63	92.88	–
Ours	EMCI61, NC161, LMCI49, AD121	dICA-LSTM	EMCI vs. LMCI	89.97	88.00	91.90	87.50	88.35
			NC vs. EMCI	91.32	91.67	91.00	90.10	89.76
			NC vs. LMCI	91.95	82.00	97.00	93.33	86.19
			AD vs. LMCI	93.53	72.14	99.17	89.06	86.27
			AD vs. EMCI	89.56	72.14	98.33	92.86	83.33
			AD vs. NC	96.14	98.13	93.53	96.88	96.73

**TABLE 6:** Comparison with other methods on ABIDE database.

References	Methods	ACC	SEN	SPE	AUC	F1
Dvornek et al. (2017)	LSTM	68.5%	–	–	–	–
Dadi et al. (2019)	Logistic regression	75.6%	–	–	–	–
Heinsfeld et al. (2018)	Two stacked denoising autoencoders	70.0%	74.0%	63.0%	–	–
El-Gazzar et al. (2019)	3D CNN-LSTM	77%	–	–	–	78%
Jiang et al. (2020)	Hi-GCN	73.1%	71.4%	74.6%	–	82.3%
Leming et al. (2020)	Ensemble deep learning	85.2%	–	–	–	92.22%
Wang et al. (2021)	CGCN	71.6%	–	–	–	–
Li et al. (2019)	GNN	76.0%	82.9%	–	0.79	–
Ours	dICA-LSTM	84.69%	98.13%	87.50%	0.89	86.05%

## Discussion

**FIGURE 7:**
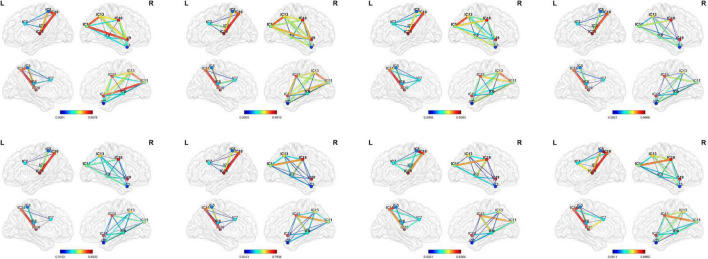
Visualization of the dynamic functional connectivity with width 50 and step size of 1. The functional connections between different resting-state networks in the sliding window are dynamic which have depth-time characteristics. (From left to right: the 10th, 20th, 30th, 40th, 50th, 60th, 70th, and 80th window).

In this study, we proposed a dynamic ICA combined with LSTM network to detect the possible dFC biomarkers from the rs-fMRI data which were applied to the disease identification aspects of AD and ASD. Specifically, the ICA was utilized to obtain maximally mutually independent states. Then, the dynamic connectivity networks were constructed from the time series of each independent component through sliding windows. The approach allowed for overlap between the time windows which had explicit temporal dynamic interpretability. Figure 7 shows the dynamic process of the brain network connectivity in AD patients in different time windows. Finally, the LSTM network with the spatial-temporal features was applied for disease diagnosis. The effectiveness of the proposed method was demonstrated by using 392 samples on the ADNI dataset (including AD, NC, EMCI, and LMCI) and 157 subjects on the ABIDE database (containing ASD and NC). These findings illustrated that the dynamic properties characterized the actual activity state better than the traditional static assumptions. This method has good performance in the application of AD and ASD. The performance of the method for other brain illnesses is not sure, and we need to do more works in the future works.

### Selection of independent components

**FIGURE 8:**
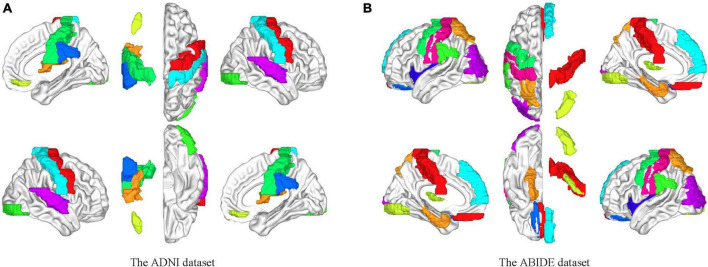
Brain mapping between the independent components and the ROIs on the AAL template. **(A)** Brain activation areas of the ADNI dataset. **(B)** Brain activation areas of the ABIDE dataset. The resting-state networks obtained by blind source analysis are mapped to the delineated ROIs, indicating the reliability of the functional networks obtained by ICA. The resting-state network ensures the homogeneity within the network and the heterogeneity between the networks compared to the specific ROI.

The independent components were usually combined and categorized into some existing specific RSN templates in the established studies, for example, the visual network, auditory network (AN), and default mode network (DMN). However, the potential discriminated information may be excluded in this process. In this study, we used all components after eliminating the obvious noisy parts. We hypothesized that there were certain subjective factors in the categorization process that could affect the experimental results. The group-level independent component decomposition method was utilized which ensured that the activation components at the same sites were obtained in all subjects and avoided the component mismatch in the individual independent component decomposition process. Figure 8 shows the correspondence brain regions between the activation components on the two datasets in this study.

### Effect of the number of components

**FIGURE 9:**
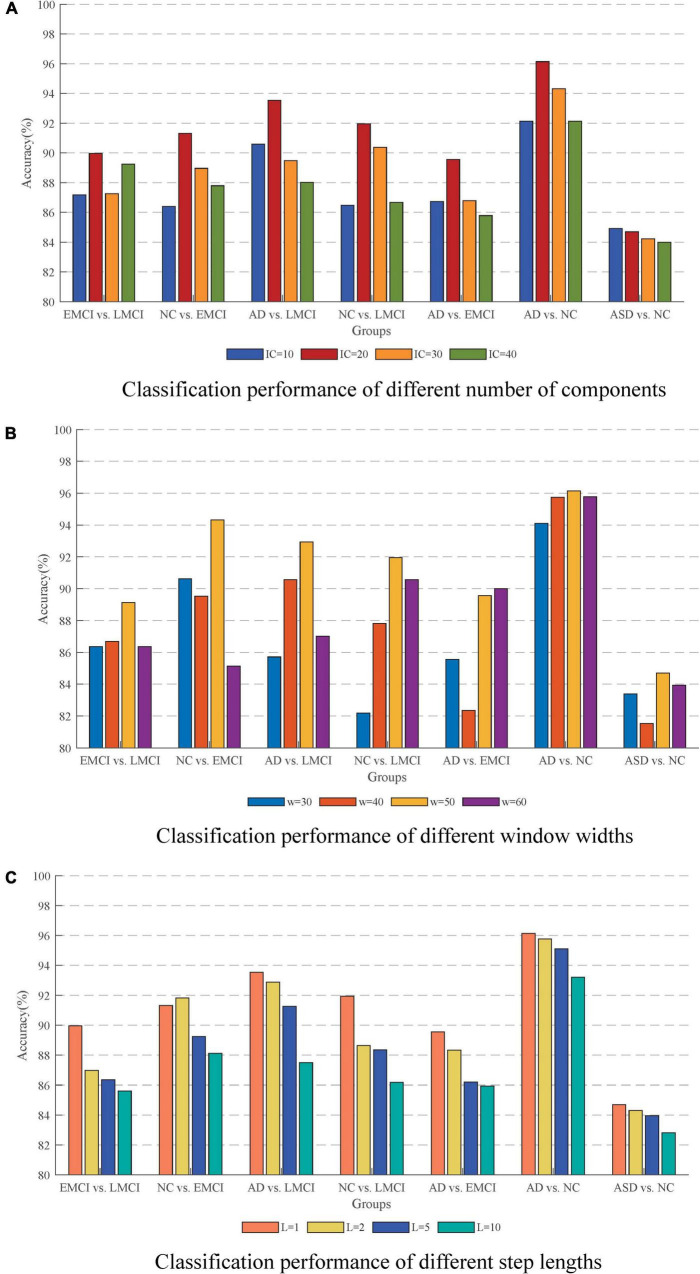
Effect of different parameters on the accuracy. **(A)** Classification performance with different number of components. **(B)** Classification performance with different window widths. **(C)** Classification performance with different step lengths. The best performance is obtained with an IC of 20, a window width of 50, and a step length of 1.

The number of independent components was one of the important factors for ICA results. The few components may result in non-independence of the ICs, while more components may lead to excessive decomposition of the independent individuals. In this study, the number of ICs was set to be 10, 20, 30, and 40 to investigate their effects on the results. Figure 9A shows the classification accuracy for each group in different cases. It could be seen that the best classification performance was obtained when the number of components was 20 which was most evident, especially in the differentiation of AD and NC.

### Effect of sliding window settings

The issue of sliding window settings of dFC networks has been widely emphasized and discussed (Preti et al., 2017; Du et al., 2020). The two most critical factors were the width of the window and the sliding step length. In this study, we conducted the classification with window widths of 30, 40, 50, and 60. The ACC results are shown in Figure 9B in which the method with window width 50 had the best performance. On the one hand, it could solve the problem of low signal-to-noise ratio that might be caused by too short a window. On the other hand, it could effectively capture the small changes of the signal in a short period of time to avoid causing information omission. For the sliding step, the step size of 1TR is suitable for the response to the temporal characteristics of the signal. As shown in Figure 9C, the temporal characteristics of the signal were lost as the step size increased.

### Effect of the long short-term memory network

The LSTM network is effective for capturing the depth features of temporal signals. In this study, we used LSTM to verify the effectiveness of dFC in fMRI data and achieved rewarding classification results. Previous studies have applied LSTM to fMRI data for disease prediction. Xing et al. (2021) proposed a graph convolution-based LSTM network for the ADNI II dataset to classify AD with NC and obtained 90% classification accuracy. Liu et al. (2020) combined multi-temporal sparse smoothing networks with LSTM for EMCI and LMCI recognition and achieved 87.93% accuracy for EMCI vs. NC and 91.49% for LMCI vs. NC. Dvornek et al. (2017) used the LSTM network to classify individuals with ASD from NC and achieved 68.5% classification accuracy. El-Gazzar et al. (2019) introduced an end-to-end 3D CNN-LSTM algorithm to extract spatio-temporal features from fMRI data which was used for the classification of ASD and controls. Another study developed a data-driven 3DConv-LSTM network model for fMRI data generation which was helpful to learn the spatio-temporal relationships of fMRI data at different time points (Zhao et al., 2020). The extensive use of LSTM in the neurological field in recent years illustrates that the network can cope well with the temporal signal of fMRI data and the weak changes in the brain over a short period of time can be used as an important feature to identify diseases. In particular, our proposed model combined LSTM with ICA to specifically depict the process of functional connectivity between spatial patterns in the brain over time. This approach further demonstrated the sensitivity of LSTM networks to temporal signals.

### Dynamic features related to Alzheimer’s disease and autism spectrum disorder

The ICA-based dynamic connectivity reflects the inherent functional interactions among different brain sub networks. The characteristic abnormal features in AD or ASD may be presented. The brain disorder-specific insights on the dynamic connectivity could serve as features for early disease classification which are helpful for the investigation of the pathogenesis.

To investigate the deep features that are related to AD or ASD, we compute the most discriminative features in the deep learning process. We found the abnormal dynamic connectivity of the DMN in AD. The abnormal dynamic connectivity between DMN and hippocampus plays a key role in the classification. The DMN is involved in the coordination of cognitive behavior. The hippocampus is inextricably linked to the production of memories. Thus, abnormal dynamic connectivity between the DMN and hippocampus may lead to decreased conscious responsiveness and memory capacity. Moreover, we found that abnormalities of the dynamic connectivity in attention network (AN) and sensorimotor network (SMN) in ASD were important for the classification. AN is responsible for perceiving external stimuli and directing attention. SMN is responsible for activity participation awareness and awakening maintenance. The anomaly of the connectivity may have contributed to the lack of awareness of participation in ASD and thus the inability to communicate properly in public places.

## Conclusion

This study proposed a dynamic ICA-LSTM approach for the fMRI disease classification which investigated the feasibility of dynamic temporal properties of functional connectivity among independent sub networks at the spatial level of the brain as potential disease biomarkers. The independent brain networks of subjects were obtained using group spatial ICA. Then, the dynamic FC features from RSNs were captured by using sliding windows. Finally, the LSTM networks were used to extract deep temporal features for disease prediction. The method exploited the dynamic temporal properties of spatial brain mapping of fMRI data by using the data-driven entry point, which solved the problem of difficulty in extracting deep temporal features by traditional machine learning methods and the self-limiting problem of model-driven. The results demonstrated that spontaneous brain activity on a time scale of seconds could be used as the distinguishable measures of brain activity.

## Data availability statement

Publicly available datasets were analyzed in this study. This data can be found here: https://ida.loni.usc.edu.

## Author contributions

JQ and ZW: conceptualization, resources, and supervision. JQ and RW: writing and methodology. RW, HL, and GX: data analysis. All authors contributed to this study and approved the submitted version.

## Footnotes

1
https://ida.loni.usc.edu
